# Valedictory

**Published:** 1883-04

**Authors:** James B. Baird


### Editorial.

### VALEDICTORY.

As previously announced, with the present issue of this journal my connection with it will cease.

It is with reluctance that I have yielded to the necessity—a necessity born of the pressing demands of imperative professional duties—of resigning a position which has afforded me so much pleasure, and which has gained for me so many agreeable associations. But there is a limit to human capacity for labor, and, notwithstanding the seductive allurements and the tempting fascinations which journalism presents, the influence of which is felt and acknowledged, the exercise of ordinary care and prudence warns me that these bounds cannot, with safety, be overreached. Submitting, therefore, to the dictates of reason I lay down the burden with a sigh of relief and regret.

The intercourse between the publisher and the editor has been uniformly the most pleasant, and its severance is not among the least regretful pertaining to this change.

The REGISTER under the fostering care, the conservative guidance and the conscientious management of my successor—Dr. John H. Logan, of this city—has my cordial good-will and sincere wishes for its continued prosperity.

With what measure of success my efforts to make it the exponent of the truest and best in the whole range of topics which it essayed to discuss I shall leave it to others to decide, and shall retire with a pleasing consciousness that to the best of my ability I have loyally labored for what I conceived to be the highest good of the medical profession.

To those kind friends who both publicly and privately have commended my editorial career I desire to return my most appreciative and heart-felt thanks. Their refreshing words of encouragement and approbation will ever be cherished among the brightest of all bright memories, and be treasured with my most valued possessions.

JAMES B. BAIRD.

